# Nanoemulgel: A Novel Nano Carrier as a Tool for Topical Drug Delivery

**DOI:** 10.3390/pharmaceutics15010164

**Published:** 2023-01-03

**Authors:** Mahipal Reddy Donthi, Siva Ram Munnangi, Kowthavarapu Venkata Krishna, Ranendra Narayan Saha, Gautam Singhvi, Sunil Kumar Dubey

**Affiliations:** 1 Department of Pharmacy, Birla Institute of Technology and Science, Pilani (BITS-PILANI), Pilani Campus, Pilani 333031, India; 2 Department of Pharmaceutics and Drug Delivery, School of Pharmacy, The University of Mississippi, Oxford, MS 38677, USA; 3 Center for Pharmacometrics and Systems Pharmacology, Department of Pharmaceutics, College of Pharmacy, University of Florida, Orlando, FL 32827, USA; 4 R&D Healthcare Division Emami Ltd., 13, BT Road, Kolkata 700056, India

**Keywords:** nano emulgel, topical delivery, permeation, surfactant, bioavailability

## Abstract

Nano-emulgel is an emerging drug delivery system intended to enhance the therapeutic profile of lipophilic drugs. Lipophilic formulations have a variety of limitations, which includes poor solubility, unpredictable absorption, and low oral bioavailability. Nano-emulgel, an amalgamated preparation of different systems aims to deal with these limitations. The novel system prepared by the incorporation of nano-emulsion into gel improves stability and enables drug delivery for both immediate and controlled release. The focus on nano-emulgel has also increased due to its ability to achieve targeted delivery, ease of application, absence of gastrointestinal degradation or the first pass metabolism, and safety profile. This review focuses on the formulation components of nano-emulgel for topical drug delivery, pharmacokinetics and safety profiles.

## 1. Introduction

**Figure 1:**
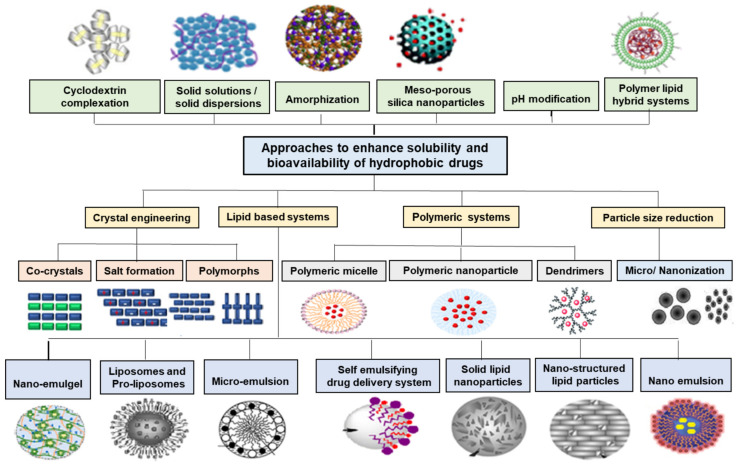
Strategies to improve solubility and bioavailability of lipophilic drugs.

The recent progress in drug synthesis and high throughput screening have steered drug discovery and development toward lipophilic drug moieties. Currently, 90% of drugs in the discovery pipeline and more than 40% of the drugs present in the market are of lipophilic nature [1]. The lipophilic nature of the drugs leads to problems like poor solubility, unpredictable absorption, and inter and intra-subject variability concerning pharmacokinetics. Various techniques have been employed to increase the solubility of active moieties. These techniques include physical and chemical modification of API along with formulation strategies, which include particle size reduction, complexation, amorphization, and nano-carrier drug delivery systems as represented in Figure 1 [2,3,4].

Despite of employing various technologies for enhancing the solubility, delivering the drugs via the oral route is not always feasible owing to their low bioavailability associated with poor absorption, first-pass metabolism, chemical and enzymatic degradation [5,6]. In addition, clinical complications and low concentrations of the drug at the site of action hinder drug delivery through the oral route. For example, the oral administration of Disease-modifying anti-rheumatic drugs (DMARDs) used in the treatment of arthritis are associated with various side effects like carcinogenicity, hepatotoxicity, and hematologic toxicity [7,8]. These clinical complications can be mitigated by delivering the drug through the topical route [9].

In topical delivery, skin being a fundamental defense layer, considers the API’s as external components and restricts their entry into the body. The outer most layer of epidermis called stratum corneum is the first and firm layer to overcome for drug penetration into the skin [10]. Various mechanisms have been explored to enhance the drug permeation. One such mechanism involves disruption of skin layer structure, which can be achieved using techniques such as chemical penetration enhancers, ultrasound, iontophoresis, sonophoresis, electroporation and microneedles [11]. In contrary, the use of nanocarriers was observed to be an effective strategy for circumventing the SC barrier without exacerbating skin damage and achieving efficient drug penetration. They facilitate the drug delivery through the skin utilizing intra and inter cellular transport mechanisms, interacting with skin components to mediate transport or to create depots of the drug for sustained or stimuli-induced release. These novel carrier for topical administration includes but not limited to emulsions (nano/micro), micelles, dendrimers, liposomes, solid lipid nanoparticles and nano-structured lipid carriers [12,13,14]. Among these, nano-emulsions are found to be a potential drug delivery system because of their high drug-loading capacities, solubilizing capacities, ease of manufacturability, stability and controlled release patterns. These nano-emulsions owing to their lipophilic core allow the movement of more lipophilic molecules across the topical membranes compared to the liposomes [15]. In addition, liposomes stability has always been an issue, as they disintegrate during the penetration process. Likewise, the low drug loading capacity and uncontrolled release hinders the application of solid-lipid nanoparticles in dermal drug delivery. Similarly, micelles exhibit poor stability and encapsulation efficiency. In the same way, the toxicity and poor controlled release behavior of dendrimers limits its topical application [16].

Nano-emulsions are heterogeneous colloidal mixtures of oil and water, with one component as a dispersed phase and the other as a continuous phase. A surfactant known as an emulsifier is adsorbed at the interface between the dispersed and continuous phases, lowering the surface tension and thus stabilizing the system. These systems possess high thermodynamic stability leading to longer shelf life compared to simple emulsions, micelles or suspensions, etc. Despite having various advantages, nano-emulsions are limited by their low viscosity leading to low retention time and spreadability [17]. These problems can be resolved by modifying the nano-emulsion into a nano-emulgel by using a suitable gelling agent [18].

The nano-emulgel acts as a colloidal system consisting of a mixture of emulsion and gel. The emulsion part protects the drug from enzymatic degradation, and hydrolysis and improves the permeation like other nano-carriers. Besides enhancing the penetration of the drug through the skin, it is equally important to retain the therapeutic concentrations of the drug for a sufficient period of time. The gel part improves the viscosity and spreadability resulting in improved retention time, and also reduces the surface and interfacial tension, thus improving the thermodynamic stability. Nano-emulgel possesses various advantages having high drug loading capacity, better penetration, diffusion, and low skin irritation compared to other nano-carriers [19,20].

This article aims to provide insight into the selection of formulation ingredients of a nano-emulgel, characteristics and formulation aspects, advantages, pharmacokinetics and pharmacodynamics, and safety of the same. The objective here is to give an overview of the future and rationale behind the nano-emulgel drug delivery system.

## 2. Drug Delivery through a Topical Route

The characteristics of any ideal formulation are patient compliance, self-administration, non-invasiveness, fewer side effects, and better pharmacological action. The topical route administering formulations possess most of the aforementioned characteristics [21]. The benefits of the topical route of administration comprise of avoiding the hepatic first-pass effect, decreased side effects due to the local site of action, enhancement in percutaneous absorption and topical usage may even increase bioavailability with a sustained deposition [22]. Further, the reduced drug loss due to metabolism or decomposition, and the ability to specifically target the drug at the desired site are also some of the advantages. Minimization of drug breakdown coupled with constant delivery of drug for a prolonged period results in prominent movement of the drug across the barrier of stratum corneum, leading to improved bioavailability [23,24].

An increase in the bioavailability of drugs via the topical route has been proved in various research works. For example, Flurbiprofen nano-emulsion showed 4.4 times increment in bioavailability upon topical administration compared to oral delivery [25]. Zhou et al. prepared the nano-emulsion of nile red dye, which displayed 10-fold increase in the penetration of dye across the skin compared to an emulsion formulation [26]. Gannu et al. reported a 3.5-fold increase in Lacidipine bioavailability via the transdermal route of administration using microemulsions. The group reported that this improvement could be due to the avoidance of the first-pass effect on the drug upon topical application [24]. Further, an enhancement in the therapeutic and pharmacological effect of therapeutically active agents has been demonstrated with topical formulations.

**Figure 2:**
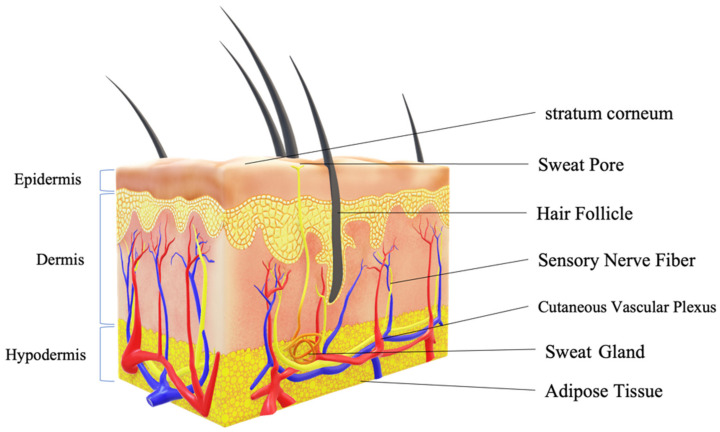
Skin morphology.

Conventional topical formulations that are used are solutions, ointments, lotions, creams, patches, gels, etc. [27]. But these topical formulations have to traverse the remarkably effective and competent stratum corneum barrier along with viable epidermis of the skin as shown in Figure 2. The SC is a 10–20 µm thick lipid-interspersed matrix of terminally formed keratinocytes, which causes a huge challenge for the delivery of therapeutically active agents via the topical or transdermal route of administration [28,29,30]. Thus, reducing the amount of drug reaching the target site. This is reflected in the marketed topical preparations possessing low permeation leading to poor therapeutic effect [31,32,33]. Therefore, research in this area mainly focuses on the development of topical formulations with appropriate permeability and ensuring delivery by numerous mechanisms. The direction of research work in recent years has shifted towards novel carrier systems with the intent to alter the permeability of hydrophobic drugs through the skin. New formulation development techniques and strategies are emerging in recent years but the main drawback of the recent strategies is the usage of chemicals and non-green solvents for enhancing the permeation. The usage of these preparations for a long period would lead to various skin complications [34,35]. Besides, various limitations posed by the skin, there are certain characteristics that an active moiety should possess in order to be suitable for the topical route of administration as represented in Table 1 [36,37].

## 3. Nano Emulsions in Topical Delivery

An upgrade and innovation of topical and transdermal drug delivery systems led to the development of lipid-based nano-formulations. Though there are various formulations, research has deepened pertaining to nano-emulsions due to the aforementioned advantages and their ability to deliver hydrophobic drugs non-invasively and without the need for a penetration enhancer [38]. Nano-emulsions are an isotropic biphasic mixture consisting of two portions: water and oil, where one phase is dispersed in the other as nanosized droplets. The system is stabilized by the utilization of an interfacial layer of surfactants [39]. The difference between nano-emulsions and traditional emulsions is that the former has decreased propensity to undergo phase separation [40,41]. A prominent number of in-vivo studies have been carried out demonstrating the applications and feasibility of topical micro and nano-emulsions. In-vitro works have also supported the use of these topical lipidic formulations [42,43]. These nano-emulsion systems possess a translucent or transparent appearance. The thermodynamic stability of nano-emulsions is greater than other lipid carriers. Nano-emulsions exhibit an increased solubilization capacity as compared to solutions of simple micelles [29,44]. These formulations can solubilize and incorporate large amounts of active drug substances due to the increased surface area because of the nano-size of oil droplets [45]. The phenomena of creaming or sedimentation are the general issues faced in an emulsion. The improved stability in a nano-emulsion is due to Brownian motion and less gravitational force acting on the particles because of their nano-size, thus preventing the stability issues like sedimentation and creaming [46]. Numerous studies have demonstrated the enhanced permeation of drugs upon administered as nano-emulsion systems in comparison to other formulations like emulsions, creams, and ointment gels [47,48,49]. The enhanced permeation is because of the ability of nano-emulsion to overcome the firmly bonded lipid bi-layers, thus able to penetrate deep into the skin and deliver the drug to systemic circulation because of smaller sized dispersed droplets, which facilitate transcellular in addition to paracellular transport [18].

## 4. Nano-Emulgel Drug Delivery System

Despite possessing many advantages, nano-emulsions lack spreadability because of their low viscosity resulting in poor retention of formulation over the skin [50]. This limitation hampers the clinical applications of nano-emulsions [51]. This issue has been resolved by incorporating a gelling agent into the nano-emulsion, thus forming a nano-emulgel [52]. Huge quantities of aqueous or hydroalcoholic bases are employed in a colloidal particulate system to prepare gels [53]. Nano-emulgel is formed by incorporating the nano-emulsion into a hydrogel matrix, which reduces the thermodynamic instability of the emulsion. The improved thermodynamic stability is due to the reduction in the portability of the non-aqueous phase because of the increased consistency of the external medium. The increased retention time and thermodynamic stability enable the formulation to release the drug over a period, making nano-emulgel a controlled release dosage form for topical administration benefiting the drugs with a short half-life [19,54].

The incorporation of nano-emulsion into a gelling system helps to annihilate the disadvantages of both individual systems. The combined nano-emulgel enjoys the properties of a gel with the refined characteristics of a nano-emulsion. The Table 2 discloses the advantages of nano-emulgel over conventional emulgel owing to its particle size and thermodynamic stability. The variety of benefits offered by nano-emulgels is enhanced skin permeation, greater loading of an active moiety, less irritation, and greater spreadability. This is apparent in comparison with different nano-carriers such as solid lipid nanoparticles and liposomes. The nano-emulsion is made suitable for topical use due to the increased viscosity of the gel. To achieve the same, various gelling agents compatible with skin like xanthan gum, carbomer 980, Pluronic’s, carrageenan, and carbomer 934 are used for topical application [55]. Acceptable localization and drug dispersion through adequate percutaneous absorption across the skin is achieved in nano-emulsions. This helps to increase the efficacy locally and also systematically via the skin. This system can also be used to deliver drugs to the central nervous system (CNS) due to its ability to cross the blood-brain barrier when applied through the nasal route [56,57]. Non-irritant and non-greasy nature of nano-emulgel facilitates better patient compliance [53] In addition, pharmacokinetic properties like enhanced bioavailability and decreased side effects are added advantages for these systems [58]. The hydrogel matrix, consistency, and homogeneity have added to the growing focus on nano-emulgels. Furthermore, various studies have shown that nanoemulgel has increased stability due to less Oswalt ripening caused by decreased mobility of oil globules in gel matrix [59]. For instance, Kaur et al. developed a topical nanoemulgel loaded with TPGS containing mefenamic acid. In the pharmacodynamic investigation, the optimized nanoemulgel inhibited inflammation and enhanced percent reaction time with improved analgesic efficacy. The formulated nanoemulgel outperformed other traditional topical formulations in terms of long-term stability and drug penetration [60].

Besides these, nano-emulgel is devoid of other formulation stability limitations like the problem of destabilization faced with conventional emulgels, the problem of moisture entrapment faced with powders, the problem of cake formation faced with suspensions, the problem of coalescence of oil globules, formation of agglomerates in case of suspensions, along with the problem of poor adherence and excessive spreadability that is faced with nano-emulsions [53]. Due to these factors, nano-emulgel is often thought of as an improved and different topical drug delivery approach over the standard marketed dosage forms. This novel formulation is welcome for research targeting various skin diseases and disorders. Nano-emulgel will soon be capturing the market in the topical delivery segment as a favorable substitute over conventional forms and some are currently being marketed as in Table 3. Many preclinical (Table 4) and clinical studies (Table 5) are being conducted to evaluate the efficacy of nanoemulgel.

## 5. Formulation Components

Nano-emulgels are made up of two individual systems; the gelling agent and the nano-emulsion i.e., emulsion consisting of nano droplets which are of o/w or w/o type. Both emulsion types possess an aqueous and an oily phase. The gel base consists of polymers that can swell on the absorption of a liquid. The various components in the nano-emulgel formulation are provided in Table 6 [53,84]. The overview of the selection criteria of the essential components in a nano-emulgel have been discussed below.

### 5.1. Oil Phase

The selection of oil and its quantity depends on the application and utility of the nano-emulgel. The permeability, stability, and viscosity of the prepared nano-emulsion depends on the type and quantity of chosen lipid component, i.e., oil phase. Primarily in case of pharmaceutical and cosmetic applications, the oil phase is made up of either naturally or synthetically originated lipids, unless the oil phase itself is an active ingredient. The consistency of the lipids may vary from liquid to high molecular solids. The hydrophobicity of an oil plays a crucial role in forming a stable emulsion, wherein poor hydrophobicity of the oil is shown to increase the emulsification, concurrently affecting the solubility of lipophilic moieties [99]. Thus, choosing an oil is an essential prerequisite for nano-emulgel development as a novel drug delivery system [100].

Natural oils exhibit an additional medicinal significance leading to an increase in the researcher’s interest to use these additive properties supporting the pharmacological action of the active moiety. For example, oleic acid is frequently used oil in nano-emulgel formulations and is obtained from vegetable and animal sources. It is a biodegradable and biocompatible omega-nine fatty acid and has elevated solubilization characteristics along with improving percutaneous absorption [101]. Antioxidants present in oleic acid contribute to cellular membrane integrity. It also repairs cell damage and showcases formulation stabilization [55,102]. Arora et al. confirmed that an increase in oleic acid content in the preparation increases the rate of permeation. In their study, using 6% oleic acid instead of 3% in the preparation nanoemulgel of drastically improved the permeability of ketoprofen [55].

Another natural oil called Emu oil is being appreciated for its analgesic, antipruritic, and antioxidant characteristics. Jeengar and group prepared nano-emulgel of curcumin with emu oil to treat the disease of joint synovium, the formulation demonstrated enhanced permeability and better pharmacological activity compared to pure curcumin [85,103]. The use of emu oil has been encouraged in the cosmetic field as well [85]. It moisturizes the skin and has high amounts of unsaturated fatty acids like oleic acid, thus improving the penetration of the drug [104].

The therapeutically active agent may also be used as the oil component in nano-emulgel preparation. Active moieties from Swietenia macrophylla have anti-inflammatory action and are self-employed as an oily phase in nano-emulgel. The therapeutic effect was found to be better in this nano-carrier preparation as opposed to the parent form [44]. Further, the edible oils considered to be the preferred lipid excipient of choice for the development of emulsions, are not frequently chosen due to their poor ability to dissolve large amounts of lipophilic drugs. Therefore, these oils are chemical modification or hydrolyzed to form an appropriate oil, which upon combining with a suitable surfactant enhances the solubility of hydrophobic compounds for nano-emulgel formulation [104].

### 5.2. Surfactant System

Surfactants are an essential ingredient in nano-emulsion, which are utilized in the stabilization of the unstable mix of two immiscible phases. This is achieved by a decreasing the interfacial tension amongst the two phases and alteration of dispersion entropy. The surfactant should show quick adsorption along the interface of the liquids. The final result is a decrease of interfacial tension and inhibition of coalescence of the individual nano-sized droplets [105].

The HLB value of the surfactant is an important variable for selecting the proper surfactant. The surfactants are either w/o type (HLB of 3–8) or o/w type (HLB of 8–16). In w/o emulsions, low HLB value surfactants i.e., less than 8 are utilized. Alternately Spans and Tweens are used for o/w emulsion as their HLB value is more than 8. A mixture of Span and Tween provides better stability to an emulsion system compared to pure Span or Tween containing preparations. Thus, using a proper mixture of surface-active agents is essential to formulate an ideal nano-emulsion. Based on the charge, the surfactants are of four main categories i.e., cationic, non-ionic, anionic, and zwitterionic nature. Examples of cationic surfactants are hexadecyl trimethyl ammonium bromide, cetyl trimethyl ammonium bromide, quaternary ammonium compounds, and dodecyl dimethyl ammonium bromide [106,107]. Poloxamer 124 and 188, Tween 20 and Caproyl 90 are some of the non-ionic surfactants [108,109]. Anionic surfactants are sodium dodecyl sulphate and sodium bis-2-ethylhexylsulfosuccinate [110]. Phospholipids such as phosphatidylcholine are part of zwitterion surfactants [111]. Toxicity should be considered while selecting the surfactant as it may lead to irritation of the gastrointestinal tract or skin based on the route of administration. Ionic surfactants are usually not preferred due to their toxicity and non-biocompatibility. The safety, biocompatibility and being unaffected by pH or ionic strength alteration make non-ionic surfactants an appropriate choice [112].

The surfactants derived from natural sources such as bacteria, fungi, and animals are being considered as a potential option, due to their safety, biodegradability, and biocompatibility. Bio-surfactants show a similar mechanism in decreasing surface tension along the interface due to amphiphilic properties. This is mainly due to the presence of non-polar short fatty acids and polar functionalities as the tail and head respectively [113]. They are more bio-compatible and safer than synthetic surfactants.

### 5.3. Co-Surfactant System

Co-surfactants support surfactants during the emulsification of oil in the water phase. Co-surfactants are required for decreasing the interfacial tension and improving the emulsification [114]. Flexibility is added to the interfacial film along with attaining transient negative interfacial tension due to co-surfactants. The association between the surfactant and co-surfactant along with the partitioning of the drug in immiscible phases decides the drug release from the nano-emulgel. Hence co-surfactant selection is equally important as surfactant. The commonly used co-surfactants are PEG- 400, transcutol^®^ HP, absolute ethyl alcohol, and carbitol [115]. Alcohol based co-surfactants are most preferred because of their ability to partition between the oil and water phase thereby improving their miscibility.

The concentration of co-surfactant being used has to be chosen cautiously, since it may affect the emulsification by surfactant. Also, a combination of surfactant and co-surfactant with closer HLB values does not produce a stable emulsion as produced by non-ionic surfactants with different HLB values. The reason may be due to the solubilization of higher HLB value surfactants in the aqueous phase. Whereas, lower HLB value surfactants solubilize in the non-aqueous phase, enabling more intense association with the mixture of surfactant and co-surfactant [116]. Therefore, the choice of various formulation components and the rationale behind them is a very demanding and stimulating exercise.

### 5.4. Gelling Agents

Gelling agents upon addition to the appropriate media as a colloidal mixture forms a weakly cohesive three-dimensional structural network with a high degree of cross-linking either physically or chemically providing consistency to nano-emulgel [117,118,119]. In topical applications, these agents are used to stabilize the formulation, to attain optimum delivery of the drug across the skin. They play an important role in determining various parameters of the formulation like consistency, rheological properties, bio-adhesive properties, pharmacokinetics, spreadability, and extrudability. Based on the origin, these gelling agents are divided into natural, synthetic, and semi-synthetic. The Table 7 gives information on the concentration and pharmaceutical adaptability of various gelling agents used to prepare nano-emulgel. Natural gelling agents are bio-polysaccharides or their derivatives and proteins. The pectin, carrageenan, alginic acid, locust bean gum, and gelatine, etc., are bio-polysaccharides, while xanthan gum, starch, dextran, and acacia gum, etc., are derivatives of bio-polysaccharides. Though they provide excellent biocompatibility and biodegradability, the major limitation of natural gelling agents is microbial degradation [119,120]. Like natural gelling agents, semisynthetic gelling agents also offer good biocompatibility and biodegradability [121]. These agents are usually the derivatives of cellulose like hydroxypropyl cellulose, ethyl cellulose, sodium alginate, etc. The semisynthetic agents are comparatively more stable than natural gelling agents and are more responsive to chemical, biological and environmental changes like pH and temperature [122]. Synthetic gelling agents are prepared by chemical synthesis, some of them are FDA-approved e.g., carbomers and poloxamers [123,124]. Carbomers are polymerized acrylic acids, while poloxamers are triblock non-iconic copolymers comprising two hydrophilic units of polyoxyethylene attached to a central hydrophobic chain of polypropylene [124,125]. The FDA-approved synthetic agents are non-toxic and offer a wide range of rheological properties based on the molecular weight of the polymer, thus suitable for a wide range of applications.

## 6. Preparation of Nano-Emulgel

Nano-emulgel is a non-equilibrium formulation of structured liquids requiring energy, surfactant, or both for its preparation. They are spontaneously formulated by mixing the components. This is undertaken by introducing energy in the biphasic system or decreasing the interfacial tension between the interfaces of the two immiscible phases [135].

**Figure 3:**
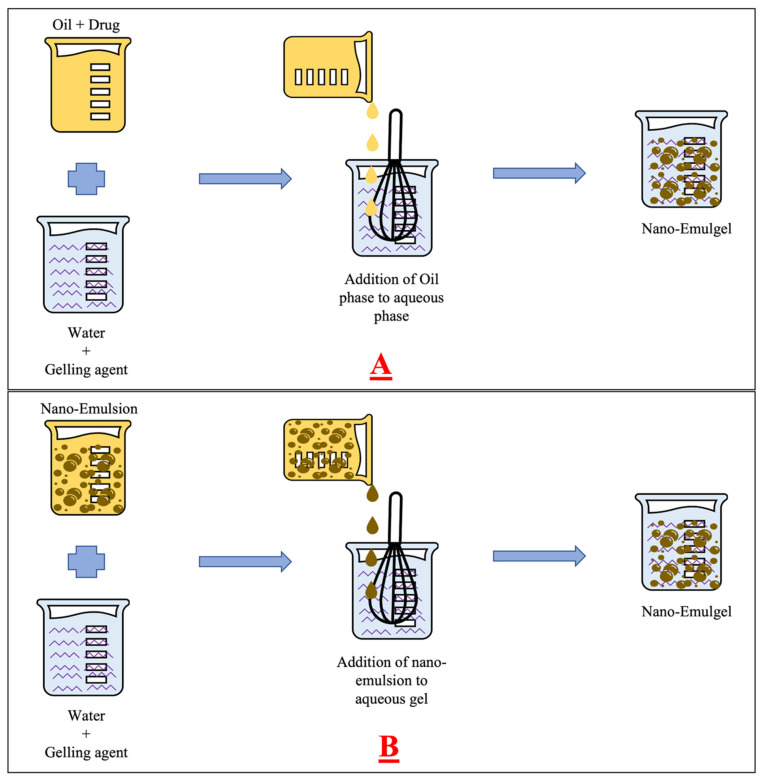
Schematic representation for the preparation of nano-emulgel by (**A**) adding Oil (oil + drug) phase to aqueous (water + gelling agent) phase (**B**) adding nano-emulsion to aqueous (water + gelling agent) phase.

There are various nano-emulgel preparation methods reported based on the order of mixing of oil and aqueous phase [136]. Lupi et al. (2014) as illustrated in Figure 3A solubilized the drug in the oil phase and gelling agent in the water phase separately. The oil phase is added to the aqueous gel phase under stirring followed by homogenization to form an emulsion. The sol form of gelling agent in the emulsion is converted to gel by various mechanisms like adding a complexing agent or adjusting to the required pH [137]. Dong et al. (2015) as illustrated in Figure 3B divided the total quantity of water required for the preparation into two parts. One part of the divided quantity is used to prepare pre-emulsion and the other part is used for the preparation of gel. Later, these two components are mixed together under stirring [138]. Jeengar et al. (2016) prepared the emulsion and gel separately, followed by mixing them together in a 1:1 *w*/*w* ratio [85].

Nano-emulgel formulation preparation can be further divided into two types based on the implementation of high-energy and low-energy emulsification techniques. High energy method involves the use of mechanical devices to produce a highly disruptive force in which both phases undergo size reduction. Hence this method may lead to the heating up of components in the formulation causing thermodynamic instability of the formulation and making it not suitable for thermo-labile drugs. Microfluidizers, high-pressure homogenizers, and ultrasonicated are high-energy methods employed to obtain a nanosized emulsion. This method is used for preparing nano-formulation of sizes of about 1 nm.

Phase inversion, self-emulsification, temperature, and phase transition are techniques of low energy approach. These methods provide the required thermodynamic stability to the nano-emulsion. The spontaneous method involves mixing oil, surfactant, and water in the best ratio possible and is most applicable for thermolabile compounds. The emulsification process is based on the surfactant and co-surfactant characteristics and their order of addition. Temperature-based alterations in HLB are utilized for non-ionic surfactants like Tween 20 Tween 60, Tween 80, Labrasol [139]. This method is mostly utilized for phase transition during phase inversion. Application of cooling with constant stirring will lead to a reversal of emulsion prepared at inversion temperature. Reduction in phase inversion temperature facilitates the inclusion of thermolabile components using this technique [140]. The second step incorporates gelling agent to change the liquid state to gel in the nano-emulsion. The thixotropic nature of the gelling agent facilitates the gel-solution conversion when shear stress is applied to the preparation keeping the volume constant. This leads to thickening in o/w nano-emulsion because of the creation of a gelled structure.

## 7. Permeability of Nano-Emulgel

In the preparation of emulsion-based gels, it is necessary to examine the important process parameters that have a significant effect on the size and formulation stability. In order to accomplish this, we must select the proper preparation process at the early stages. Emulsions are developed using various techniques, such as mechanical (or rotor-stator), high-pressure, microfluidization, and ultrasonic methods. The mechanical system comprises a colloid mill, that has a complex geometry, and the droplets of an emulsion generated by this system are several microns in size, making it the least desirable approach for manufacturing nanoemulsions [141]. Achieving an optimum droplet size is highly challenging. However, a droplet size of less than a micron can be achieved using high-pressure homogenization and sonication techniques, which in turn helps extend the shelf life of emulsions by lowering the creaming rate. For this reason, homogenization and sonication are considered to be efficient methods for the development of nanoemulsion [142,143]. In addition, increasing the homogenization speed or duration by itself is not enough to decrease the size of the globules, however, the use of the optimum concentration of an emulsifier is necessary to maintain control over the re-coalescence of the emulsion. For instance, Sabna Kotta et al. made a nanoemulsion utilizing the phase inversion and homogenization methods. In this formulation, gelucire 44/14 was used as a surfactant and transcutol-HP as a co-surfactant. They employed both the proposed techniques to produce nano-sized emulsion globules. In the case of homogenization, the large globule size was observed, despite increased pressure, and increased cycles at lower concentrations of an emulsifier. This demonstrates that the globule size of the formulation could not be decreased by homogenization alone. When the optimum concentration of an emulsifier is combined with increasing homogenization pressure and cycles, the size of the globules decreases. Because homogenization alone can break down globule size to nano, but with a lower concentration of surfactant, the newly formed globule surface would be improperly covered with a surfactant, resulting in re-coalescence. With the optimum concentration of an emulsifier and increased homogenization pressure and cycles, a smaller globule size with a good polydispersity index could be achieved. As a result, the author came to the conclusion that, throughout the preparation process, the desired particle size was obtained with a lower PDI by the combination of the surfactant, homogenization pressure, and cycle duration [142].

Mohammed S. et al. used ultrasonication to develop a thymoquinone-loaded topical nanoemulgel for wound healing. They used black seed oil (oil vehicle), Kolliphor El (surfactant), and Transcutol HP (co-surfactant). Nanoemulgel was prepared using different time intervals (3, 5, and 10 min) of ultrasonication at a 40% amplitude. When the concentration of surfactant decreased with 10 min of ultrasonication, the globular size increased. Meanwhile, increasing the concentration of surfactant with 10 min of sonication time resulted in a smaller globular size. The authors concluded that sonication is more effective when the appropriate concentration is used [70]. Monitoring the process control parameters and taking into account the composition of the excipients is both necessary steps in the process of optimizing the formulation.

## 8. Permeability of Nano-Emulgel

**Figure 4:**
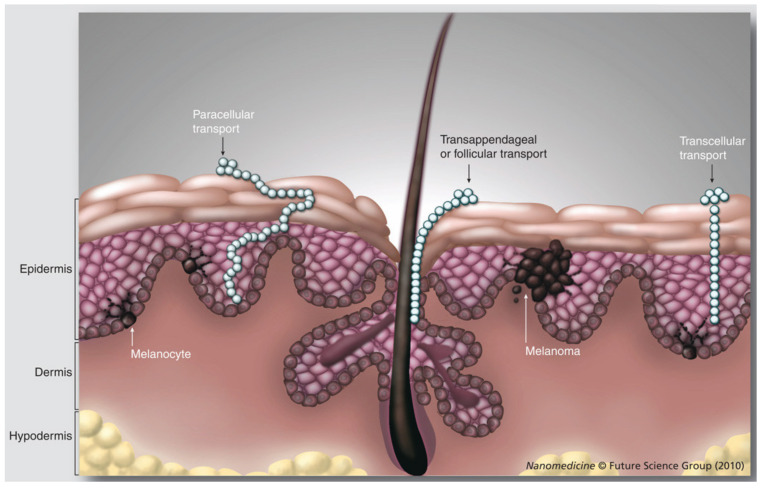
Graphical representation of entry of nano-emulgel into skin [144]. Adapted from Nanomedicine, 3 December 2010; 5(9): 1385–1399. Copyright (2010) Future Science Group.

Skin shows an inherent property of acting as a protective barrier against external agents. Therefore, penetration through the skin is a major complication associated with topical delivery systems. The outermost layer of skin is the stratum corneum, which is followed by stratum granulosum and stratum lucidum. The stratum corneum is loosely composed of keratinized cells, waxy lipids, fatty acids, and cholesterol. All these constituents of stratum corneum help in retaining moisture and provide a hydrophobic barrier over the skin [18]. After the stratum corneum, there is the epidermis which is followed by dermis and subcutaneous layer. After crossing the subcutaneous layer, the active moiety will finally reach the systemic circulation. The primary hurdle for the drug moiety after reaching out from gel matrix is crossing the stratum corneum, from here the nano sized droplet due to the virtue of small diameter traverses basically through two different pathways as shown in Figure 4. One is cell to cell transfer involving concentration gradient-based movement called transcellular transport or intracellar transport, while the other is a passage through intercellular spaces or paracellular transport [118]. Whereas there is a third pathways called transappendageal transport, its influence on drug penetration is limited because hair follicles and glandular ducts make up negligible portion of the total surface area of the skin [16].

Generally, ex-vivo permeation studies involve the examination of nano-emulgel formulation on isolated tissue in a simulated biological medium. Ex-vivo studies give a comparative analysis of penetration with different types of topical dosage forms and an idea about the flux rate of the drug inside the skin. Jeengar et al. made a nano-emulsion with emu oil as the oil phase. Optimized nanoemulsion was amalgamated with Carbopol gel to form nano emulgel and used for topical delivery of curcumin as an anti-inflammatory agent in rheumatoid arthritis. Ex-vivo permeation studies showed that permeation through the skin was higher for nano-emulgel as the retention of the formulation was higher compared to the nano-emulsion [145,146]. Elmateeshy and group formulated nano-emulgel by incorporating terbinafine HCl (TB) nano-emulsion formed from peceol as oil phase and (TWEEN 80/propanolol) as surfactant mixture and Carbopol as a gelling agent. The enhanced permeation of peceol oil-based nano-emulgel was observed in ex-vivo studies compared to available marketed products [97]. Similarly, Mulia et al. developed nano-emulgel for mangosteen extract composed of o/w nano-emulsion with virgin coconut oil as the oil phase and Tween 80/SPAN 80 as surfactant mixtures. The gel base was made with xanthan gum and phenoxyethanol was supplemented as a preservative. In vitro permeation studies demonstrated elevated penetration compared to nano-emulsion [29,147,148]. In addition, Bhattacharya et al., formulated celecoxib nano-emulgel with carbopol-940 hydrogel base, while Tween 80 and Acconon MC8-2EP as surfactants. Both in-vitro drug release and ex-vivo studies showed positive results. After the twelfth hour of diffusion, the optimized formulation displayed 95.5% cumulative release of the drug, whereas the commercially available formulation showed only 56.90% release. A higher penetration coefficient is displayed by nano-emulgel compared to commercial formulation [149]. In the same way, Chin et al. also developed a nano-emulgel of telmisartan for intranasal delivery using different molecular weight chitosan polymers. The ex-vivo penetration studies showed an improved permeation profile. The group also demonstrated the improvement in permeation is attributed to the molecular weight of the polymer, where the medium molecular weight chitosan provides higher permeation [150].

A nano-emulgel preparation for delivery through the transdermal route of tacrolimus was formulated by Begur et al. using almond gum as gel and oleic acid as the lipophilic phase. Cremophor was used as a surfactant to improve penetration. Examinations on rat abdominal skin showed a substantial increase in penetration [34]. Similarly, butenafine an antifungal agent available as a cream in the market, Syamala et al. prepared a nano-emulgel formulation for the same drug and found considerable results. Ex-vivo penetration studies showed a substantial increase in permeation over marketed creams [97]. In the same way, an increment in permeation of ketoconazole by about 53% was observed by delivering the drug in nano-emulgel formulation compared to normal marketed cream. The quality of life of the patient could be improved by implementing these types of dosage forms [151]. These studies showcase the ability of nano-emulgel in enhancing the permeation of the active moiety compared to nano-emulsion and conventional topical dosage forms. The permeation of the nano-emulgel is affected by various factors like gelling agents, surfactants, and permeation enhancers, etc. The gelling agents improve the permeation by improving the adherence of formulation upon the skin. While the surfactant alone or in combination with a co-surfactant will improve the permeation by disrupting the lipid bilayer. All these components can improve the permeation of active moiety.

## 9. Characterization Studies of Nano-Emulgel

The pharmaceutical product must be evaluated to ensure quality and consistency between different batches. These tests help in understanding the product’s behavior and stability. According to USP, there are few universal tests for any given dosage form e.g., description, identification, assay, and impurities. A topical dosage form should undergo a few specific tests set by USP on a case-by-case basis: uniformity of dosage units, water content, microbial limits, antimicrobial and antioxidant content, pH, particle size, sterility, and API’s polymorphic nature. Apart from the tests required for topical dosage form, nanoemulgel consists of nanosized globules, which need to be evaluated for zeta potential, droplet size and polydispersity index (PDI). Along with these physiochemical tests, a dosage form needs to be evaluated for its in-vitro release, spreadability, bio-adhesive tests, skin-irritation, ex-vivo permeability and in-vivo bioavailability can be performed to understand the behavior of nanoemulgel. Methods and techniques for analyzing significant properties of a nanoemulgel are briefly described below:

### 9.1. Zeta Potential

The particles in a solution usually possess a layer of ions on their surface, referred to as the stern layer. Adjacent to the stern layer, there exists a diffuse layer of loosely bounded ions, which along with the stern layer collectively called an electrical double layer. There is a boundary between the ions in the diffuse layer that move with the particle and the ions that remain with the bulk dispersant. The zeta potential is the electrostatic potential at this “slipping plane” boundary [152]. Zeta potential measurement provides an indirect measure of the net charge and is a tool to compare batch-to-batch consistency. The higher the zeta potential, the greater the repulsion resulting in increased stability of the formulation. For example, the high zeta potential of emulsion globules prevents them from coalescing. A surface charge modifier may also be used to adjust the surface charge. For instance, if a negatively charged surface modifier is used, the zeta-potential value becomes negative, and vice-versa [153,154]. Surface active ingredients (such as anionic or cationic surfactants) thus play an important role in emulsion stability, and the zeta potential can be measured using various instruments such as the ZC-2000 (Zeecom-2000, Microtec Co. Ltd., Chiba, Japan), Malvern Nanosizer/Zetasizer^®^ nano-ZS ZEN 3600 (Malvern Instruments, Westborough, MA, USA), and others.

### 9.2. Droplet Size Measurement and Polydispersity Index (PDI)

The size of globule in nanoemulgel is referred as its hydrodynamic diameter, which is a diameter of equivalent hard sphere that diffuses at the same rate as the active moiety [155]. The PDI determines the distribution of droplet size and is defined as the standard deviation of droplet size divided by mean droplet size. The droplet size and the polydispersity index are closely connected to the stability and drug release, as well as the ex-vivo and in-vivo performance of the dosage form. In addition, it is important to measure consistency between different batches. The globule size and PDI of the formulation can be measured using a zeta sizer or master sizer. The globule size of the emulsion can be determined using the principle of dynamic light scattering, in which the transitional diffusion coefficient is measured by monitoring the interaction between the laser beam and dispersion, as well as the Polydispersity index [156,157].

### 9.3. Rheological Characterizations

Rheology is the study of the deformation and flow of materials. The rheological characterization of materials reveals the influence of excipient concentrations like oils, surfactants, and gelling agents on the formulation’s viscoelastic flow behavior. If a formulation’s viscosity and flow characteristics vary, this may influence its stability, drug release, and other in-vivo parameters. In this instance, the formulation’s shear thinning tendency generates a thin layer on the skin surface, improving permeability, whereas a thicker formulation decreases permeation. Therefore, the rheological behavior is an extremely important factor in the formulation of nanoemulgel and several unique types of viscometers can be used to determine the rheological behavior [20]. FDA recommends the evaluation of complete flow curves whenever possible, plotted as both heat stress versus shear rate and viscosity versus shear rate across multiple shear rates until low or high plateaus are observed. If a formulation exhibits plastic flow, yield stress values should be evaluated.

### 9.4. Spreadability Testing

The spreadability property of the topical dosage form ensures the evenly spreading of the dosage form, thus delivering a stranded dose subsequently affecting the efficacy. The viscosity of the nanoemulgel greatly affects the spreadability property [158]. To date, no standard method has been established for measuring the spreadability of the dosage form. A few tests, that are commonly used for a good approximation of spreadability are a parallel-plate method and human subject assessment etc. The parallel-plate method (slip and drag method) is a widely employed technique because of its simplicity and relatively economic [158]. The instrumental setup consists of two glass slides of the same length, one of which is stationarily attached to the wooden block, and the other glass slide is mobile attached to a pulley at one end to measure spreadability. Spreadability is determined by the emulgel’s ‘Slip’ and ‘Drag’ qualities. The nanoemulgel dosage form will be placed on a stationary glass slide, which is then squeezed in between stationary and mobile glass slides. The formulation is squeezed firmly for uniformly spreading formulation between two slides and to remove any air bubbles. The known weights are added to the pulley until the upper slide slips off from the lower slide. The time required for slipping off is recorded, which is used to calculate spreadability using the following equation [159].

S=M∗L/T

where, *S*, *M*, *L* and *T* respectively represent the spreadability, weight bounded to the upper slide, Length of the slide, and Time taken to detach the slides.

### 9.5. In-Vitro Release Test (IVRT)

The efficacy and safety of the API are associated with drug release from the dosage form. The IVRT serves as a tool for assessing the quality of the drug product [160]. According to FDA, the IVRT studies for semi-solid dosage forms are conducted using either the vertical diffusion cell or an immersion cell. The vertical diffusion cell consists of receptor and donor chambers, separated by a receptor membrane. The donor chamber holds the sample of dosage form, while the receptor chamber holds the receptor media. The receptor media can be a buffer or hydro-alcoholic solution, selected based on the solubility, sink condition, and stability of the API. The skin-like receptor membrane is selected based on the effective pore size, high permeability and expected inertness towards the API. If necessary, the receptor membrane should be saturated with release media. The temperature of the media should be maintained around 32 °C ± 1 °C for topical administering products, for products intended for mucosal membrane the temperature should be 37 °C ± 1 °C. A Teflon-coated magnetic stirrer is used for stirring the receptor media. While the immersion cell model has a cell body, which acts as a reservoir [161]. The cell body is covered with a membrane and closed using a leakproof seal (retaining ring cap) that ensures no leakage of the dosage form. The retaining ring cap possesses an opening on the top, and it should be adjusted in such a way that the membrane is in contact with the dosage form on the bottom and release media on the top. The whole setup is used along with the USP-2 apparatus, wherein the immersion cell is placed in flat bottomed dissolution vessel with a usual volume of 150–200 mL. A mini spin-paddle is used for stirring or agitating the media [162].

### 9.6. Bio-Adhesive Property

Bio-adhesive strength is used to determine the force required to detach the drug carrier system from a biological surface. This property is important for a topical dosage form if prolonged contact is required [163]. This test is usually performed using rat or pig skin, the latter is preferred because of its resemblance to human skin. There are various techniques to measure this property but none of them is approved by FDA. The texture analyzer is one such technique, where the upper mobile probe and stationary lower base plate will be covered with skin. The dosage form is placed on the skin of the base plate. The upper probe is lowered to contact the lower base plate and the contact is maintained for at least a minute. The upper probe is lifted slowly until the separation of skin sheets. The force required to separate the two skin sheets will be measured by the instrument and represented as the area under the force-distance curve [164].

## 10. Safety Issues

One of the crucial concerns while developing a skin-related formulation is toxicity and skin irritation [165]. Impairment of enzyme activity, disturbance in normal physiological functions, and sometimes carcinogenic effects (For e.g., being caused by Sodium do-decyl benzene sulfonate) are some common toxicity issues related to surfactants [166]. Smith and the group analyzed the effect of two surface active agents’ sodium dodecyl sulfate and dodecyl trimethyl ammonium bromide on penetration and skin perturbation. They concluded that disruption in the layer of skin is primarily caused by elevated concentrations of micelle agglomerate and monomers [167].

Irritation caused by topical nano-emulgel can be examined by applying it on the shaven skin of a rat, then observation of redness and other signs of inflammation on the skin were made and then graded based on the number of eurythmic spots to assess their clinical implication as give in Table 8 [167]. In general, a grade scale up to 2 is safe. Azeem et al. prepared ropinirole nano-emulgel using caproyl 90, tween 20 and carbital. The skin irritation studies showed a grade 2 erythema index, which is safe [168]. Gannu et al. also performed skin irritation studies of nano-emulgel prepared using non-ionic surfactant the Tween 80 and co-surfactant labrasol. They observed no signs of skin irritation as the used surfactants are generally considered safe [25]. Usually, major toxic effects are observed with cationic surfactants so they are avoided in preparations associated with topical delivery. While nonionic surfactants are mostly preferred as they cause minimum perturbation of the skin layer [151,166].

## 11. Challenges

The impartment of large drug entities with molecular weight exceeding 400 Dalton is hindered in this dosage form, as they show difficulty during size reduction and are found to leach out of the gel mesh network. A limited number of safe surfactants and co-surfactants are available for emulgel preparation. Not much maneuvering can be done with the selection of surfactant as it can have hazardous consequences. The abundance of surfactant in emulgel can lead to skin problems like contact dermatitis, erythema, redness of skin, skin layer perturbation [169]. High susceptibility of the gelling agent toward variations in pH and temperature can lead to the breaking of gel structure and the leaching of chemicals [170].

Capriciousness in nano-emulsion is caused due to Ostwald ripening, which is associated with nano size of oil droplets, preferably nano-emulsion is prepared shortly before its application. Optimizing the speed of the stirrer in the homogenizer (as required to produce an inflexible and non-cracking gel), mixing appropriate quantities of surface-active agents, and selecting a reliable packing material are very pivotal tasks associated with the stability of nano-emulgel [171]. Highly specialized instruments are required for size reduction to nanoscale, which requires handling by skilled labour. Expensive sustenance of high energy homogenizers and production cost is one of the critical limitations associated with scale up of nano-emulgel formulation. Besides these disadvantages, the comforting prospect of nano-emulgel is increased adherent property and elevated embranglement of the drug in the gel mesh [53]. Also, prevalent drawbacks associated with conventional topical dosage forms i.e., emulsion, ointment, lotions etc. such as creaming, phase disruption, oxidation induced degradation of ointments are overcome by forming emulgel [44].

## 12. Current and Future Prospects of Nanoemulgel

Delivering hydrophobic drugs to the biological systems has been a major challenge in formulation development owing to their low solubility, leading to poor bioavailability. Some of the topical formulations include creams, ointments, and lotions. They possess good emollient characteristics, however, has slow drug release kinetics due to the presence of hydrophobic oleaginous bases such as petrolatum, beeswax, and vegetable oils, which inhibit the incorporation of water or aqueous phase. On contrary, topical aqueous-based formulations like gels enhance the drug release from the medication since it provides an aqueous environment for medicament. Therefore, hydrophobic APIs are blended with oily bases to form an emulgel, which further undergoes nanonization to form a nanoemulgel with enhanced properties. The superior properties of a nanoemulgel like thermodynamic stability, permeation enhancement, and sustained release make it an excellent dosage form. There are several marketed emulgels and patents being filed (Table 9) for the same, demonstrating its tremendous progress in this field. By making advancements in the ongoing research, nanoemulgel, as a delivery system would outshine, in formulating the drugs that are being eliminated from the development pipeline owing to their poor bioavailability, therapeutic non-efficacy, etc. Despite these advantages, the manufacturing of nano-emulsion limits its commercialization. However, with the progressing technology, commercially feasible and profitable manufacturing techniques could be possible in the future. With the advantages of nano-emulgel over other formulations, a tremendous increase in the production of nano-emulgel can be foreseen.

## 13. Conclusions

The selection of ingredients and their appropriate ratios play a vital role in deciding the properties of a nano-emulgel. Deviation from this could affect the conversion of a nano-emulsion to a nano-emulgel and its thermodynamic stability. The nano-emulgel is more stable compared to that of a nano-emulsion mainly due to its less mobile dispersed phase and the decreased interfacial tension. Thus, the former is a better alternative in delivering lipophilic moieties mainly due to improved permeation, and better pharmacokinetics, which subsequently improves the pharmacological effect. Patient compliance is also elevated due to its non-greasy and improved spreading properties on topical administration. Despite of its advantages, nano-emulgel is still at its infancy in the prospect of the pharmaceutical industry. However, various emulgels are being marketed e.g., Voltron emulgel, which holds out hope for the commercialization of nano-emulgel in near future. Hence it has the potential to become a center of attention due to its safety, efficacy, and user-friendly nature for topical drug delivery. Despite some disadvantages, nano-emulgel is a tool for the future which may be an alternative to traditional formulations.

## Figures and Tables

**Table 1:** Primary requirement of active moiety for topical delivery.

Properties	Conditions
t_1/2_	≤10 h
Molecular mass	≤500 Daltons The limit can be exceeded by altering the permeability of skin
Molecular size	Small
Polarity	Non-polar is desirable
Log P	0.8–5
pKa	Higher
Irritation on skin	Non-irritating
Skin Permeability coefficient	≥0.5 × 10^–3^ cm/h

**Table 2:** Comparison between conventional emulgel and nano-emulgel.

Parameter	Conventional Emulgel	Nano-Emulgel
Thermodynamic stability	Not stable because of natural tendence of coalescence leading to sedimentation or creaming [61]	Stable–because of their smaller particle size, Brownian motion provides enough stability against gravity, preventing sedimentation or creaming [54]
Particle size	Greater than >500 nm [18]	Less than 100 nm [62]
Bioavailability	Comparatively less bioavailable than Nano-emulgel [63]	Enhanced bioavailability, attributed to small size and large surface area [64]
Permeation	Comparatively lower permeation [65]	High permeation owing to its lower particle size [54,65]
Preparation	Require high energy techniques [66]	It can be prepared either by using high or low energy techniques [20]
Systemic absorption	Very minimal	Higher compared to conventional emulgel due to the small particle size and large surface area [54]
Ability to cross BBB	Cannot cross BBB [67]	Can Cross BBB because of its small particle size [68]

**Table 3:** Examples of marketed emulgels for topical application.

Marketed Product	Active Pharmaceutical Ingredient	Manufacturing Company
Voltaren Emulgel	Diclofenac diethylamine	GlaxoSmithKline
Isofen Emulgel	Ibuprofen	Beit Jala Pharmaceutical Co.
Benzolait Emulgel	Benzoyl peroxide & Biguanide	Roydermal
Miconaz-H Emulgel	Miconazole nitrate & Hydrocartisone	Medical Union Pharmaceuticals
Derma Feet	Urea	Herbitas
Adwiflam Emulgel	Diclofenac diethylamine, Methyl Salicylate & Menthol	Saja Pharmaceuticals
Nucoxia Emulgel	Etoricoxib	Zydus Cadila Healthcare LTD

**Table 4:** Pre-clinical Studies on the nano-emulgel dosage form.

Active Ingredient	Composition	In Vivo Model	Route of Administration	Therapeutic Outcome	Reference
Curcumin	Oil: Labrofac PG + transcutol HP Surfactant mixture: Tween 20 + solutol HS15 Gelling agent: Carbopol 934	BALB/c mice	Topical	Psoriatic mice treated with the curcumin nano-emulgel showed faster and earlier healing than those treated with curcumin plus betamethasone-17-valerate gel	[69]
Thymoquinone	Oil: Black seed oil Surfactant mixture: Kolliphor EL + transcutol HP Gelling agent: Carbopol 940	Wistar rat	Topical	Nano-emulgel administration of thymoquinone improves its therapeutic efficiency in wound healing studies in Wistar rats	[70]
Curcumin and Resveratrol	Oil: Labrofac PG Surfactant mixture: Tween 80 Gelling agent: Carbopol	Wistar rat	Topical	Curcumin and resveratrol nano-emulgel technology revealed drastically increased curcumin and resveratrol deposition in skin layers. The in-vivo investigation revealed that the NEG formulation resulted in improved burn healing, with histological findings comparable to standard control skin. Thymoquinone nano-emulgel delivery method improves thymoquinone therapeutic effectiveness in wound healing studies in Wistar rats.	[71]
Brucine	Oil: Myrrh oil Surfactant mixture: Tween 80 + PEG 400 Gelling agent: Carboxymethylcellulose sodium	BALB/c mice and Wistar rats	Topical	Brucine-loaded nanoemulgel has shown improved anti-inflammatory and anti-nociceptive efficacy.	[72]
Curcumin	Oil: Labrofac PG Surfactant mixture: Tween 80 + PEG 400 Gelling agent: Carbopol 940	Albino rats	Topical	Curcumin nanoemulgel improved the wound-healing efficacy of curcumin compared to the conventional gel formulation.	[69]
Raloxifene hydrochloride	Oil: Peceol Surfactant mixture: Tween 20 + transcutol HP Gelling agent: Chitosan	Wistar rats	Topical	Raloxifene hydrochloride (RH) loaded nanoemulgel formulation for enhanced bioavailability and anti-anti-osteoporotic efficacy of RH. The bioavailability improved by 26-fold compared oral marketed product.	[73]
Eprinomectin	Oil: Castor oil Surfactant mixture: Tween 80 + Labrasol Gelling agent: Carbomer 940-1	Wistar rats	Topical	Naoemulgel formulation showed improved skin permeability of 1.45-fold compared to emulgel and had no skin-irritating property	[74]
Amisulpride	Oil: Maisine CC Surfactant mixture: Labrosol + transcutol HP Gelling agent: Poloxamer 407, Gellan gum	Wistar rats	Intranasal	Improved pharmacokinetic profile. The C_max_ of API in brain after administering through in-situ nano-emulgel improved by 3.39-fold compared to intravenous administration of nano-emulsion.	[75]
Disulfiram	Oil: Ethyl oleate Surfactant mixture: Tween 80 + transcutol HP Gelling agent: Deacetylated gellan gum	Sprague Dawley rats	Intranasal	Improved survival rate of rats and reduced tumor progression (Glioblastoma). The survival time of in-situ nano-emulgel treated group is 1.6 times higher than control group	[76]

**Table 5:** Clinical studies on emulgel dosage form.

Identifier No	Active Constituent	Titile of the Study	Conditions	Referance
NCT05536193	Metformin and salicylic acid	Topical Metformin Emulgel VS Salicylic Acid Peeling in Treatment of Acne Vulgaris	Acne Vulgaris	[77]
NCT03074162	Diclofenac sodium & Capsaicin	Comparison of the Bioavailability of Diclofenac in a Combination Product (Diclofenac 2% + Capsaicin 0.075% Topical Gel) With Two Diclofenac Only Products, Diclofenac Mono Gel 2% and Voltarol^®^ 12 Hour Emulgel 2.32% Gel, in Healthy Volunteers	Inflammatory	[78]
NCT04579991	Visnadin, ethyl ximeninate, coleus barbatus	Effects of Visnadin, Ethyl Ximeninate, Coleus Barbatus and Millet in Emulgel on Sexual Function in Postmenopausal Women	Female Sexual FunctionVulvovaginal AtrophyPostmenopausal Atrophic Vaginitis	[79]
NCT04110860	Voriconazole	Clinical Assessment of Voriconazole Self Nano Emulsifying Drug Delivery System Intermediate Gel	Tinea Versicolor	[80]
NCT04110834	Itraconazole	Clinical Assessment of Itraconazole Self Nano Emulsifying Drug Delivery System Intermediate Gel	Tinea Versicolor	[81]
NCT03492541	Propylene glycol-based eye drops	Evaluation of the Clinical Efficacy and Tolerability of SYSTANE Complete in Adult Patients With Dry Eye Disease Following Topical Ocular Use for 4 Weeks: A Multicenter Trial	Dry eye disease	[82]
NCT05641246	Carbamide diclofenac	Effect of Topical Diclofenac on Clinical Outcome in Breast Cancer Patients Treated With Capecitabine: A Randomized Controlled Trial.	Hand and Foot Syndrome	[83]

**Table 6:** Details of commonly used excipients in nano-emulgel formulations.

S.No	Disease/ Disorder	Active Pharmaceutical Ingredient	Composition				References
			Oil	Surfactant	Co-Surfactant	Gelling Agent	
1	Anti-inflammatory	Curcumin	Emu oil	Cremophor RH40	Labrafil M2125CS	Carbopol	[85]
2	Anti-inflammatory	Diclofenac sodium	Isopropyl myristate	Tween 20	Labrafil M2125CS	Carbopol 980	[86]
3	Anti-inflammatory	Meloxicam	Almond and peppermint oil (1:2)	Tween 80	Ethanol	Carbopol 940	[87]
4	Antimicrobial and Anti- Inflammatory	Quercetin	Cinnamon oil	Tween 80	Carbitol	Poloxamer	[88]
5	Antifungal	Itraconazole	Eugenol	Labrasol	TranscutolP, Lecithin	Carbolpol	[89]
6	Antifungal	Fluconazole	Capmul MCM	Tween 80	Transcutol P	Carbopol 934	[90]
7	Anti-hyperglycemic	Glibenclamide	Labrafac: Triacetin (1:1)	Tween 80	Diethylene glycol monoethyl ether	Carbopol 934	[91]
8	Antihypertensive	Carvedilol	Oleic acid: IPM (3:1)	Tween 20	Carbitol	Carbopol-934	[92]
9	Immunosuppressive agent	Cyclosporine	Oleic acid	Tween 80	Transcutol P	Guar gum.	[93]
10	Anti-cancer	Chrysin	Capryol 90	Tween 80	Transcutol HP	Pluronic F127	[94]
11	Wound Healing	Atorvastatin Calcium	Liquid Paraffin	Tween 80	Propylene glycol	Sodium carboxymethyl cellulose	[95]
12	Anti-inflammatory	Curcumin	Myrrh Oil	Tween 80	Ethanol	Sodium carboxymethyl cellulose	[96]
13	Wound Healing	Curcumin	Labrofac PG	Tween 80	Propylene glycol 400	Carbopol 940	[69]
14	Anti-fungal	Terbinafine HCl	Peceol oil	Tween 80	Propanol	Carbopol 940	[97]
15	Anti-fungal	Ebselen	Captex	Kolliphor ELP	Dimethylacetamide	Soluphus (10% *w*/*v*) & HPMC K4M (2.5% *w*/*v*)	[98]

**Table 7:** Various gelling agents and their pharmaceutical adaptability for use in topical emulgel.

Gelling Agent	Concentration Range (%*w*/*w*)	Pharmaceutical Adaptability	Reference
HPMC	2–6%	Forms neutral gels Can provide good stability Resists microbial growth	[126,127]
Carbomer (Carbopol) Grades–ETD 2020, 171, 910, 934, 934P, 940, 1342 NF, 1971P	0.1–1.5%	Forms high viscous gel Forms gel at very low concentration Provides controlled releasep H dependent gelling	[126,128]
NaCMC	3–6%	It withstands autoclaving. Therefore, can be used in sterile gels Stable between pH 2 to 10	[129,130]
Poloxamer Grades–124, 182, 188, 407	20–30%	Possess better solubility in cold water Thermoreverisble gelation–gel at room temperature and liquid at refrigerated conditions	[131,132]
Combination of HPMC & Carbopol	1.2%	Combination can improve stability of emulsion compared to individual components	[133,134]

**Table 8:** Skin irritation grading scale and their clinical implications.

Clinical Portrayal	Grade
No erythema	0
Slight erythema that is barely perceptible	1
Moderate erythema that is visible	2
Erythema and papules	3
Severe Edema	4
Erythema, edema, and papules	5
Vesicular eruption	6
Strong reaction spreading beyond the application sight	7

**Table 9:** Recent patents on nano-emulgel.

Patent Number	API	Title	Disease Indication	Current Assignee/Inventors	Granted/Publication Year	Reference
US11185504B2	Aromatase inhibitors	Transdermal non-aqueous nanoemulgels for systemic delivery of aromatase inhibitor	breast cancer	Qatar University	2021	[172]
CA3050535C	Anti-inflammatory nutraceuticals e.g., resveratrol, cinnamaldehyde, green tea polyphenols, lipoic acid etc.	Methods of treating inflammatory disorders and global inflammation with compositions comprising phospholipid nanoparticle encapsulations of anti-inflammatory nutraceuticals	Inflammatory Disorders	Nanosphere Health Sciences Inc	2021	[173]
CN107303263B	Tripterygium glycosides	Tripterygium glycosides nanoemulsion gel and preparation method thereof	Immune diseases e.g., clinical rheumatoid arthritis and psoriasis etc.	Second Military Medical University SMMU	2020	[174]
EP3099301B1	Besifloxacin	Besifloxacin for the treatment of resistant acne	Acne vulgaris	Vyome Therapeutics Ltd.	2019	[175]
WO2020240451A1	Brinzolamide	In-situ gelling nanoemulsion of brinzolamide	glaucoma	Hemant Hanumant BHALERAO, Sajeev Chandran	2020	[176]
WO2020121329A1	Minoxidil and castor oil	Minoxidil and castor oil nanoemulgel for alopecia	androgenic alopecia	Sudha suresh Dr. Rathodsoniya ramesh devasani	2020	[177]
BR102019014044A2	Ketoconazole	Nanoemulgel based on ucúuba fat (*Virola surinamensis*) for transungual administration of antimicotics	Onychomycosis	Rayanne Rocha Pereira et al.	2021	[178]
